# Intelligent diagnosis and prediction of pregnancy induced hypertension in obstetrics and gynecology teaching by integrating GA

**DOI:** 10.3389/fmed.2024.1433479

**Published:** 2025-02-12

**Authors:** Xiaolan Li, Fen Kang, Xiaojing Li

**Affiliations:** ^1^Department of Obstetrics and Gynecology, The First Affiliated Hospital of Anhui Medical University, Hefei, China; ^2^NHC Key Laboratory of Study on Abnormal Gametes and Reproductive Tract (Anhui Medical University), Hefei, China; ^3^Key Laboratory of Population Health Across Life Cycle (Anhui Medical University), Ministry of Education of the People’s Republic of China, Hefei, China; ^4^Anhui Province Key Laboratory of Reproductive Health and Genetics, Hefei, China; ^5^Anhui Provincial Engineering Research Center of Biopreservation and Artificial Organs, Hefei, China

**Keywords:** obstetrics and gynecology teaching, genetic algorithm, pregnancy induced hypertension, intelligent diagnosis, feature selection

## Abstract

**Title:**

Advanced Diagnosis and Forecasting of Pregnancy-Induced Hypertension in Obstetrics and Gynecology Education through the Integration of Genetic Algorithms.

**Background:**

Pregnancy-induced hypertension represents a critical issue within the fields of obstetrics and gynecology, where precise diagnosis and forecasting are essential for effective management. The potential for misdiagnosis, often stemming from the inexperience of healthcare professionals, underscores the necessity for an advanced diagnostic system.

**Methods:**

This research introduces an innovative sampling and feature selection technique grounded in *F*-scores optimization, alongside the development of a comprehensive prediction model that integrates genetic algorithms with various heterogeneous learners. The objective of this model is to maximize the utility of medical data and enhance treatment quality.

**Results:**

The refined intelligent feature selection approach identified several significant indicators of pregnancy-related hypertension, such as phosphor dehydrogenase deficiency, body mass index, gestational urinary proteins, vascular endothelial growth factor receptor 1, placental growth factor, thalassemia, and a familial history of diabetes mellitus or hypertension. The model achieved superior performance metrics, including the highest recall (0.768), *F*-score (0.728), and area under the curve (0.832) when compared to other prevalent models. Furthermore, the area under the curve for both early and late clinical assessments reached peak values of 0.996 and 0.792, respectively, when evaluated using the ratio of vascular endothelial growth factor receptor 1 to placental growth factor.

**Conclusion:**

The intelligent diagnosis and prediction methodology for gestational hypertension proposed in this study exhibited remarkable efficacy and holds significant promise for implementation in both educational and clinical settings within obstetrics and gynecology, thereby advancing intelligent medical diagnostics in China.

## 1 Introduction

The medical industry has seen a significant change due to the swift advancement of artificial intelligence. Obstetrics and gynecology, a crucial area of medicine, has played a major role in bringing about this change (1). Among them, intelligent medical diagnostics (IMD), prediction, and decision-making performance improvement are not only conducive to the effectiveness and efficiency of the treatment of diseases but also helps to reduce the pressure of hospitals and the psychological burden of patients (2). However, the current method of using intelligent medical devices for examination and relevant professionals to develop appropriate treatment programs through their own experience and knowledge is influenced by human factors, and it relies on the knowledge level of the decision maker, which has greater limitations (3, 4). Furthermore, one of the main causes of maternal and perinatal mortality is pregnancy-induced hypertension (PIH), which is the coexistence of increased blood pressure during pregnancy. PIH can have a substantial negative impact on the health of mothers and infants (5). According to the data, the prevalence of PIH disease in China is increasing, but the cause and mechanism of the disease are still unclear, and it may be related to mother age, physical condition, family history, and other factors (6). Elevated blood pressure can cause insufficient cerebral blood supply, heart failure, and other conditions, which are not conducive to the development of brain health. It also leads to spasm of small arteries in the brain, which induces the manifestation of ischemia and hypoxia, maternal headache and dizziness, and other symptoms (7, 8). In newborns, PIH leads to insufficient oxygen supply to the placenta, making it difficult for the newborn to obtain adequate nutrients in the maternal uterus, resulting in growth restriction and delays. In severe cases, developmental delay is the consequence. The paper initially suggests using the hybrid sampling technique known as AMOM-DUMS, which combines the artificial minority oversampling method (AMOM) with deleted undersampled mixed sampling (DUMS) to address the aforementioned issues. The integrated prediction of integrating multiple heterogeneous learners with GA (IPIMHL-GA) is also constructed. The study aims to solve the problem of unbalanced medical data classification, design intelligent PIH diagnosis and prediction methods applicable to obstetrics and gynecology teaching (OGT) and clinics, and advance the development of intelligent medical diagnosis. The research has three primary innovations. The first point is to propose intelligent AMOM-DUMS sampling algorithm to reduce the noisy data generated during the sampling process. The second point is to design the intelligent medical data feature selection (FS) method with *F*-score optimization to solve the problem of feature redundancy. Building the IPIMHL-GA model is the third step in enhancing the model’s prediction capabilities.

This approach uses the advantages of both techniques to enhance the model’s performance. Specifically, the study employs *F*-score optimization for feature selection, which effectively identifies the most pertinent features for predicting PIH. Furthermore, it utilizes the IPIMHL-GA classification algorithm, which amalgamates the capabilities of various heterogeneous learners to boost the accuracy of PIH predictions. The research also incorporates a hybrid model that synergizes different methodologies to further enhance prediction accuracy that has been supported by a comprehensive dataset that facilitates the assessment of the model across a wide range of scenarios. This work not only aims to refine the accuracy of PIH predictions but also introduces a novel evaluation metric, such as the *F*-score, to assess model performance. The innovative aspects of this research hold the promise of improving PIH prediction accuracy, thereby potentially lowering healthcare costs, enhancing patient care, and contributing to advancements in medical research. By offering a more precise predictive model for PIH, the study could lead to improved patient outcomes and a reduction in morbidity and mortality. It also paved the way for new insights and discoveries within the field.

The study is organized into four primary sections. The review of relevant research findings is in the first section. The intelligent medical data sample and FS technique design, which is based on *F*-score optimization and the IPIMHL-GA model, is covered in the second section. The performance and clinical application impacts of the study’s suggested approaches are validated in the third section. Eventually, conclusion is in the last section.

## 2 Related work

Under the background of digitalization wave that is sweeping the world, the deep integration of artificial intelligence technology and medical field, and with the advantages of precision, high efficiency and wide range of applications gradually reshape the form and efficiency of medical services and medical teaching to realize precision medicine. Numerous scholars have conducted in-depth analysis and discussion on this issue. Deng et al. (9) proposed an intelligent jaundice medical diagnosis system based on dynamic uncertainty causality diagrams (DICD) to address the imbalance of medical resources and other deficiencies in China’s healthcare system. A DICD knowledge base was constructed for jaundice diagnosis. In that study, 203 jaundice-related medical records were randomly selected from 3,985 medical records in the hospital for testing, and the accuracy of the system’s final diagnosis reached 99.01%. Real-time reverse transcription polymerase chain reaction testing is currently a popular method for detecting the New Crown Pneumonia Virus, but it has some limitations, including a lengthy turnaround time and a significant risk of false positive and false negative results. Therefore, Saurabh et al. (10) designed an integrated intelligent and automated diagnostic system. The outcomes demonstrated that the system may successfully increase the diagnostic procedure’s speed and efficiency. Tian et al. (11) aimed to develop and validate a clinical characterization and intelligent hypoxic-ischemic brain injury recognition model based on conventional structural magnetic resonance imaging. The study collected full-term hypoxic-ischemic brain injury neonates and healthy neonatal controls from December 2015–2020 from two different medical centers. The research findings demonstrated that the intelligent diagnosis (ID) model outperformed the basic radiomics model in terms of discriminatory ability, with area under the curve (AUC) values in the training, internal validation, and independent validation cohorts of 0.868, 0.813, and 0.798, respectively. Early identification of hepatitis C virus disease has long been a focus of medical research and a significant public health concern. Accordingly, Terlapu et al. (12) built five machine learning models using probabilistic neural network-based techniques. The experimental results revealed that the random forest (RF) maximum likelihood model outperformed other traditional maximum likelihood algorithms with an accuracy of 97.5%, and the proposed model based on incremental hidden layer neurons had an accuracy of 99.6%. All the models constructed in the study outperformed the base model, while early hepatitis C virus diagnosis was relevant to the work and medical field.

In the medical field, the data classification imbalance problem is a pervasive phenomenon. However, if the data category imbalance is not solved, the direct construction of the machine learning model may yield overly optimistic or even useless classification results. Consequently, numerous scholars have devoted considerable attention to the data classification imbalance problem and the associated processing techniques. Barot and Jethva (13) addressed the problem that data imbalance makes it difficult for classification algorithms to achieve the desired performance goals; moreover, the minority-sensitive Naive Bayes (NB) algorithm was designed to eliminate the requirement of dataset sampling, and the study compared it with cost-sensitive techniques. The results indicated that the algorithm proposed by the study showed more excellent results for unbalanced data classification with improved prediction performance for few categories of data. In semiconductor manufacturing that is highly precise and comprises numerous unit processes, even minor defects can have significant consequences, directly impacting the result. The monitoring of equipment condition and the study of possible problem causes are made possible by fault detection and classification, and these activities are critical to resolve the issue of imbalanced data in mass production. Therefore, Kim et al. (14) proposed a stepwise fault diagnosis method with fault detection and fault classification. The findings of the exercise demonstrated that the research raised hair functions effectively in real-world settings and could also give engineers part-level notifications. Zhang et al. (15) designed an improved EEG classification algorithm for the problem of unbalanced medical data classification. Among the triple classification results, the research method achieved an average recall of 51.56%, providing an alternative method for recognizing targets based on a fast serial visual presentation paradigm. Although it has not gotten much attention in DensePose, data imbalance has grown to be a significant challenge to current techniques. In order to solve this problem, Sun et al. discovered the DensePose mechanism underlying intra- and inter-surface imbalance and created a technique that combined block balance localization with adaptive equalization loss. The study technique outperformed the base method’s baseline by 1.4 AP, according to experimental results on the DensePose-COCO dataset (16).

The current findings highlighted the need for a more balanced approach to the prioritizing of intelligent medical diagnosis and data categorization, as demonstrated by the preceding research. However, the existing research has not fully addressed the nuances of the distribution of medical data features in terms of their relative importance. Concerning the above problems, the research gets optimized by three perspectives, including sampling, FS and, model, and an intelligent medical data sampling and FS method have been proposed based on *F*-score optimization as well as the IPIMHL-GA model.

## 3 Design of diagnostic and predictive methods for PIH

The application of imbalanced data in IMD and prediction is very extensive, but the current processing techniques of data categorization imbalance usually generate noise, which results in poor classification and prediction of intelligent methods. Therefore, the study proposes an intelligent medical data sampling and FS method based on *F*-score optimization and designs an intelligent PIH diagnosis and prediction method by incorporating GA for OGT, i.e., IPIMHL-GA model.

### 3.1 Feature selection method for smart medical data based on *F*-score optimization

With the increasing abundance of data in the medical field, the problem of data imbalance becomes more serious, which brings great challenges to IMD and decision making. It also increases the complexity of the application of intelligent methods in OGT (17). Therefore, the study addresses the problem of medical data imbalance, and the study takes PIH imbalanced data as the research object. Firstly, the imbalanced dataset is quantified by unbalanced ratio (UR), and then the FS method is used to help the related personnel to filter the features with the highest relevance in order to get the models with strong predictive ability and judgment markers to advance the process of precision medicine. Furthermore, the study optimizes the approach of data classification imbalance from the standpoint of sample size and suggests an algorithm for sampling processing called AMOM-DUMS. The used PIH dataset contains (A) hyperbilirubinemia data, (B) preeclampsia data, and (C) preterm infant data. These the data are divided into two categories, including majority and minority, which are recorded as 1 and 0, respectively. In dataset A, the normal range of concentrations is between 1.7–17.1 μmol/L, which is recorded as 1, and the other values of concentrations are recorded as 0 (18). In dataset B, systolic/diastolic blood pressure greater than or equal to 160 mmHg/110 mmHg, more than 3 + random urinary proteins, or more than 5 g of 24-h urinary protein content are recorded as 1, and the rest of the data are recorded as 0. In dataset C, neonates with gestational age less than 37 weeks are recorded as 0, and the rest of the data are recorded as 1 (19–21). This brings up the PIH dataset’s details, which are displayed in Table 1.

**Table 1:** Characteristics of the pregnancy-induced hypertension dataset.

Data set	A	B	C
Number of samples for “0”	392	86	122
Number of samples for “1”	1,695	2,008	1,958
UR	4.324	23.349	16.049

**Figure 1:**
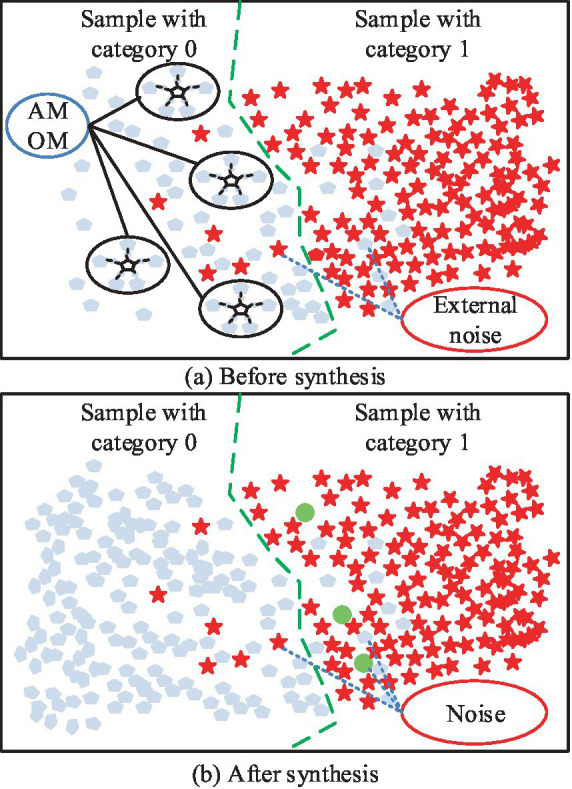
Schematic diagram of AMOM method processing flow: **(a)** Schematic diagram of binary classification before processing; **(b)** Schematic diagram of binary classification after AMOM method processing.

Since the medical data are generally out of balance, the study uses the AMOM-DUMS algorithm for processing. The specific procedure in the AMOM stage is as follows. Firstly, let the sample marked as “0” be $u_{i}$, and search for the nearest m samples. Secondly, randomly select sample $u_{ij}$ from the searched samples, and take a random number s between 0 and 1. Then, calculate the difference sample of feature j. Finally, a new synthetic sample $u_{\mathrm{new}}$ is obtained, which is added to the dataset and noted as a sample belonging to 0 (22, 23). The flow of processing using the AMOM method is shown in Figure 1.

**Figure 2:**
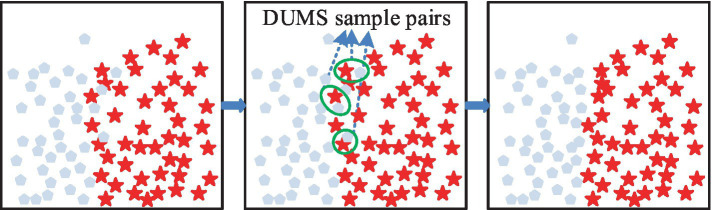
Schematic representation of the delete undersampled mixed sampling.

In Figure 1a, the five closest similar samples belonging to “0” are selected, and the corresponding Euclidean distances are multiplied by s to obtain $u_{\mathrm{new}}$, and the above steps are repeated. Figure 1b shows the final result; the number of samples belonging to “0” is obviously increased, but it also generates noisy data, which cannot be processed by the AMOM method. The exact processing flow is depicted in Figure 2. The study uses the DUMS approach to filter the noisy samples in order to improve the classifier’s classification performance by removing surrounding examples of various classes.

**Figure 3:**
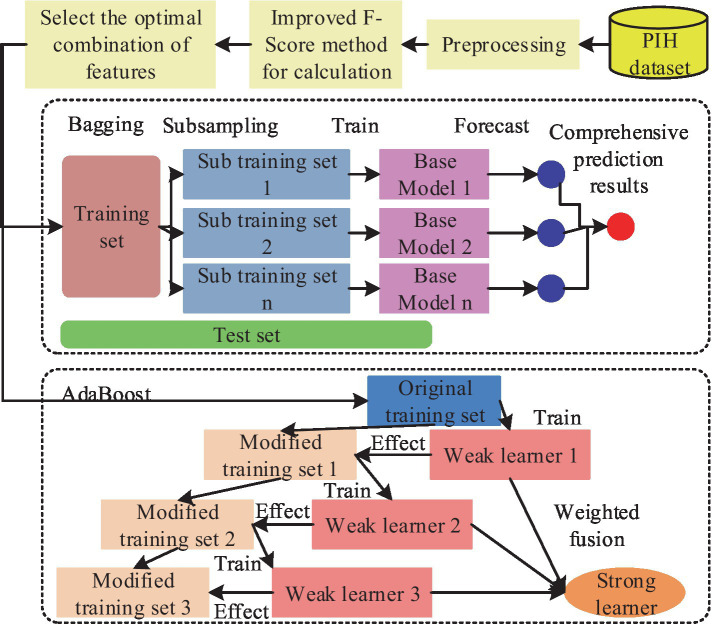
Flowchart of the enhanced *F*-score feature selection algorithm for optimal classification model performance in pregnancy-induced hypertension diagnosis.

In Figure 2, if there is a difference between categories and neighboring samples with one step distance, then there is a DUMS between the samples, and the samples with closer distance need to be removed. The above operation must be repeated until all the samples with DUMS, where closer distance exists, are removed (24, 25). After the above data sampling is completed, it can be fed into support vector machine (SVM) to balance the classification error with the complexity of the prediction model, the expression is shown in Equation 1 (26).


(1) $$\min_{\omega ,b,\alpha }\frac{1}{2}\omega^{T}\omega +\beta \overset{n}{\underset{i}{\sum }}\beta_{i}\geq 1-\beta_{i},\beta_{i}\geq 0,i=1,2,\ldots ,n$$

where, ω denotes the normal vector of the hyperplane, and b, $v_{i}$, α and $\beta_{i}$ correspond to the bias term, output label, penalty coefficients, and slack variables, respectively. After the above sampling process is completed, FS can be performed. First, data are collected from pregnant women who gave birth in hospitals and are singletons. Also, patients with multiple pregnancies, untreated endocrine disorders, diagnosed PIH or diabetes, and severe liver and kidney disease, and cardiovascular disease are excluded. This resulted in a PIH-1 dataset with a UR of 50.86, a sample size of 42 and 2,136 for 0 and 1, respectively, and a feature size of 25. The features with large missing values in the dataset are then deleted along with postpartum fasting insulin, gestational diabetes mellitus treatment, cesarean section, time to preterm premature rupture of membranes, postpartum glycosylated hemoglobin, and umbilical vein blood pH. Among other things, the study newly constructed several characteristics, like weight during pregnancy, calculated by the difference between the weight at delivery and the weight before pregnancy. The date of the last menstrual period and the date of delivery are added with 1–4 eigenvalues, corresponding to the seasons of spring, summer, fall and winter. Body mass index (BMI) has been calculated by the ratio of weight to height squared (27, 28). Because of the noise, heterogeneity, and complexity of the collected data, the study first invited obstetrician and gynecologist to screen the variables, namely laboratory data, basic information features and clinical variables. Then the features with large missing values are removed and normalized to improve the training efficiency. The calculation is shown in Equation 2.


(2) $$u\left(i\right)=\frac{f\left(i\right)-f_{\text{min}}\left(i\right)}{f_{\text{max}}\left(i\right)-f_{\text{min}}\left(i\right)}$$

where, $f\left(i\right)$ is the feature corresponding to $u\left(i\right)$, $f_{\text{min}}\left(i\right)$, and $f_{\text{max}}\left(i\right)$ are the minimum and maximum values of $f\left(i\right)$, respectively. Following the aforementioned processing, the *F*-score of each feature in the initial feature set is measured using the conventional *F*-score algorithm to determine the effective feature subset. Its application in the binary classification problem is more effective, and the specific process is as follows. Firstly, assuming that the training sample is $f\left(i\right)$, the *F*-score $F\left(i\right)$ of the ith feature is calculated, and the expression is shown in Equation 3.


(3) $$F\left(i\right)=\frac{{\left({\bar{U}}_{i}^{+}-{\bar{U}}_{i}\right)}^{2}+{\left({\bar{U}}_{i}^{-}-{\bar{U}}_{i}\right)}^{2}}{\frac{1}{n_{+}-1}\sum_{a=1}^{n_{+}}{\left({\bar{U}}_{a,i}^{+}-{\bar{U}}_{i}^{+}\right)}^{2}+\frac{1}{n_{-}-1}\sum_{a=1}^{n_{-}}{\left({\bar{U}}_{a,i}^{-}-{\bar{U}}_{i}^{-}\right)}^{2}}$$

where, $n_{+}$ and $n_{-}$ correspond to the data samples of positive and negative categories. ${\bar{U}}_{i}$, ${\bar{U}}_{i}^{+}$ and ${\bar{U}}_{i}^{-}$ are the datasets for the ith feature, respectively. $n_{+}$ and $n_{-}$ correspond to the mean values, and ${\bar{U}}_{a,i}^{+}$ and ${\bar{U}}_{a,i}^{-}$ correspond to the ith feature corresponding to the ath $n_{+}$ and $n_{-}$ referring to the eigenvalues, respectively. Nonetheless, the study enhances recognition, when the ratio of positive to negative class samples is unbalanced, as the typical *F*-score algorithm handles the few sample attributes. This is done in a way that the PIH samples with the value of 0 are $n_{-}$, while the samples belonging to 1 are designated as $n_{+}$. This results in the improved *F*-score value $F^{\prime }\left(i\right)$ expression, as shown in Equation 4.


(4) $$F^{\prime }\left(i\right)=\frac{{\left({\bar{U}}_{i}^{+}-{\bar{U}}_{i}\right)}^{2}+{\left({\bar{U}}_{i}^{-}-{\bar{U}}_{i}\right)}^{2}}{\frac{1}{n_{+}-1}\sum_{a=1}^{n_{+}}{\left({\bar{U}}_{a,i}^{+}-{\bar{U}}_{i}^{+}\right)}^{2}}$$

where, the larger the feature of $F^{\prime }\left(i\right)$, the stronger the ability is to recognize a smaller number of sample groups. To explore the classification effect of the improved *F*-score algorithm in the prediction model, the study designed the corresponding classification model, which is shown in Figure 3.

In Figure 3, it is necessary to sample the unbalanced data of medicine, then the $F^{\prime }\left(i\right)$ of the features should be calculated and sorted, then the best combination of the acquired features must be input into the classifier. The data is also trained by AdaBoost model and bagging model, finally the results without FS are compared with the predicted results (29, 30). Among the aforementioned models, the AdaBoost model is notable for its ability to employ the outputs of multiple weak classifiers to enhance the model’s generalizability and predictive performance. This is achieved through a visual representation of the weak classifiers, which elucidates the analytical and decision-making processes. Furthermore, the model’s trustworthiness is enhanced, leading to greater confidence in the intelligent method among related people. The $G\left(u\right)$ calculation is presented in Equation 5.


(5) $$G\left(u\right)=\overset{K}{\underset{k=1}{\sum }}w_{k}f_{k}\left(u\right)$$

where, K is the weak classifiers, and $f_{k}\left(u\right)$ and $w_{k}$ correspond to the kth weak classifier and its weight. By synthesizing the above formula, the intelligent medical classification model based on the improved *F*-score algorithm can be obtained.

### 3.2 Diagnosis and prediction of PIH with GA fusion

**Figure 4:**
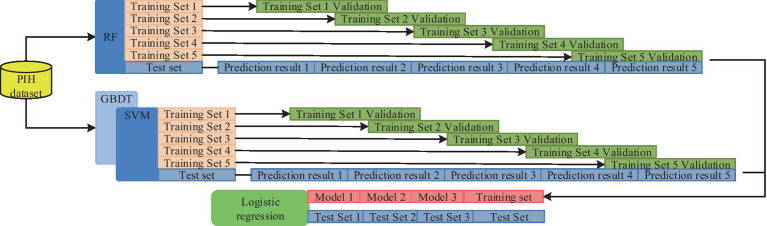
The whole framework of the integrated learning optimization model.

The research develops an IPIMHL-GA model, which is primarily composed of the integrated learning optimization model and the GA, in order to further optimize the performance of IMD-oriented and prediction models, enhance the models’ capacity for learning, and simplify the intelligent method’s operation. The whole framework of the integrated learning optimization model has been represented in Figure 4.

In Figure 4, the first layer has 10 classifiers, such as SVM and AdaBoost. This setup enables several weak classification sets to become strong classifiers and avoids the variance problem arising from a single classification model. The training set is then divided based on cross-validation and trained with several base classifiers as well as prediction using the validation set. Finally, the prediction results of all the subsets are merged to obtain a new training set to be transferred to the second layer for further training. The study uses a regression logic approach to assess the fusion of the results of the first layer, and the final result can be obtained. The calculation is shown in Equation 6.


(6) $$P\left(v=1|u\right)=\frac{1}{1+e^{-{w^{\prime }}^{T}u}}$$

**Figure 5:**
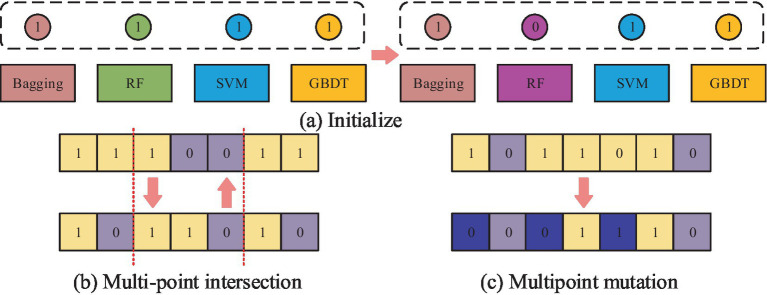
The operational flow of each part of the GA for adapting ensemble learning optimization models: **(a)** Initialization operation process; **(b)** Multi point cross operation process; **(c)** Multi point mutation operation process.

In Equation 6, v and $w^{\prime }$ are the classification labels and the weight parameters of the model, respectively. The metaclassifier of regression logic approach not only adapts to various types and sizes of data but also has good generalization ability and robustness. The parameters of the model are adjusted using regularization methods to avoid underfitting or overfitting problems. In the GA section, the study designed a GA adapted to the integrated learning optimization model, which included initialization operation, crossover, variation and selection, and the flow. This procedure is shown in Figure 5.

Figure 5a shows the initialization operation process, which needs to initialize each classifier into an individual with length of 10, which has the value between 0 and 1. Then, a number of individuals are randomly generated to form a population, where the number 0 represents the deletion of the corresponding classification model. If it is 1, it means that the corresponding classification model is retained and applied in integrated learning. Figure 5b shows the process of multipoint crossover operation, which requires the model combination corresponding to the gene strings to set multiple crossover points to swap gene blocks. It is possible to create new persons by fusing the gene blocks of several individuals, which increases population category diversity and prevents the issue of local optimal solutions. The algorithm’s convergence time can be slashed by using this technique to mix the genes of the best individuals to produce kids that perform better. The multipoint mutation operation is illustrated in Figure 5c. Its goal is to improve the algorithm’s global search capabilities by randomly selecting various locations on the genes to expand the variety of the search space through mutation. After the completion of each of the above operations, the individuals suitable for the next generation can be screened by the roulette wheel selection method. This is done by calculating the likelihood of each individual appearing in the offspring based on the fitness value of the individual and randomly selecting individuals to form the offspring population according to the probability. This not only retains the better performing individuals but also avoids situations, such as unfairness or bias in the algorithm’s selection operation. The roulette selection method is calculated in Equation 7.


(7) $$P_{i}=\frac{{\mathrm{fit}}_{i}}{\sum_{j=1}^{N}{\mathrm{fit}}_{j}}$$

In Equation 7, N denotes the population size, ${\mathrm{fit}}_{i}$ and $P_{i}$ are the fitness value corresponding to individual i and the probability of being selected, respectively. The specific steps of selection are explained subsequently. Firstly, a random probability value must be obtained. Secondly, the selection probability of individuals must be accumulated, then there should be a search for the first one so that the accumulated probability exceeds the random probability value. After that, the accumulated probability must be selected. The above process must be repeated until sufficient individuals are obtained and. In addition, the fitness value selects the exact rate acc for calculation as in Equation 8.


(8) $${\mathrm{fit}}_{i}=\frac{{acc}_{i}}{\sum_{j=1}^{N}{acc}_{j}}$$

Since PIH data has the imbalance property, i.e., the negative samples will far exceed the positive samples, if the threshold value is too high, then the predicted samples will all be negative samples, and the acc-value will be high, but the actual classification effect is opposite. Therefore, the study uses the AUC value of receiver operating characteristic (ROC) as the fitness function of the algorithm and the predicted value. According to the GA, it is possible to obtain the best performing classifier combination for the first layer of the integrated learning optimization model, i.e., AdaBoost with Gaussian process (GP), and the former expression is shown in Equation 9.


(9) $$D_{z}\left(u\right)=D_{z-1}\left(u\right)+\chi_{z}d_{z}\left(u\right)$$

where, $D_{z}\left(u\right)$ is the previous weighted cumulative results of the z base classifiers, and $\chi_{z}$ and $d_{z}\left(u\right)$ are the weights and predictions of the zth base classifier, respectively. In addition to preventing the PIH prediction from overfitting, the model can modify the weights of the training samples such that the base classifiers focus more on the incorrectly classified data. The expression of GP classifier is shown in Equation 10.


(10) $$P\left(v_{i}=1|u_{i},X\right)=\zeta \frac{y_{i}^{T}{\left(Y+\psi^{2I}\right)}^{-y}}{\sqrt{1+\psi^{2y_{i}^{T}}{\left(Y+\psi^{2I}\right)}^{-y}}}$$

where, X, ζ, Y, and $\psi^{2}$ are the training dataset, the cumulative distribution function of the standard normal distribution, the kernel matrix, and the noise variance, respectively. $y_{i}$ represents the similarity vector of the ith sample and all the samples in X. The GP classification method is able to learn by the labeling information and features of the samples in X, and the method is also used to predict the samples in the validation set. Finally, the hyperparameters of the integrated learning optimization model are intelligently tuned using the grid search method. All hyperparameter combinations are enumerated at the same time to realize the global search for the optimal solution in the hyperparameter space, and the specific process is shown in Equation 11.


(11) $$\left\{ \begin{array}{l} h_{1}:\left[c_{11},c_{12},\ldots ,c_{1n_{1}}\right]\\ h_{2}:\left[c_{21},c_{22},\ldots ,c_{2n_{1}}\right]\\ \ldots \ldots \\ h_{y}:\left[c_{y1},c_{y2},\ldots ,c_{yn_{y}}\right] \end{array}\right.$$

**Figure 6:**
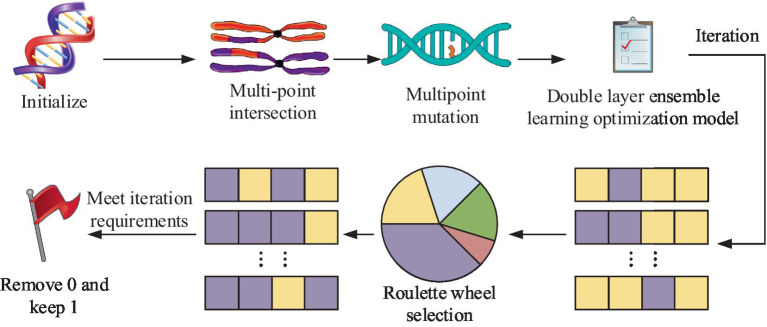
Schematic diagram of the framework of the IPIMHL-GA model.

In Equation 11, $h_{i}$ and $n_{i}$ are the number of ith hyperparameter and its candidate values, respectively, and $c_{ij}$ stands for hyperparameter of jth candidate value. Finally, the combination of hyperparameters with the best performance index effect can be obtained. The complete IPIMHL-GA model can be obtained by synthesizing the above equation, and the specific framework is shown in Figure 6.

In Figure 6, the study uses the *F*-score optimization method for FS operation, initialization operation, crossover, mutation and selection of the integrated learning model optimized by GA, and iteration to get the prediction result with the best effect.

## 4 Results of intelligent PIH diagnostic and predictive methods

To examine the performance of ID and prediction methods for OGT and the effect of clinical application, the study first explores the performance of the underlying *F*-score optimized intelligent data sampling and FS methods, then it analyzes the performance of the IPIMHL-GA model. In the end, it applies the intelligent methods in clinical trials.

### 4.1 Performance analysis of *F*-score optimized feature selection method for smart data

**Figure 7:**
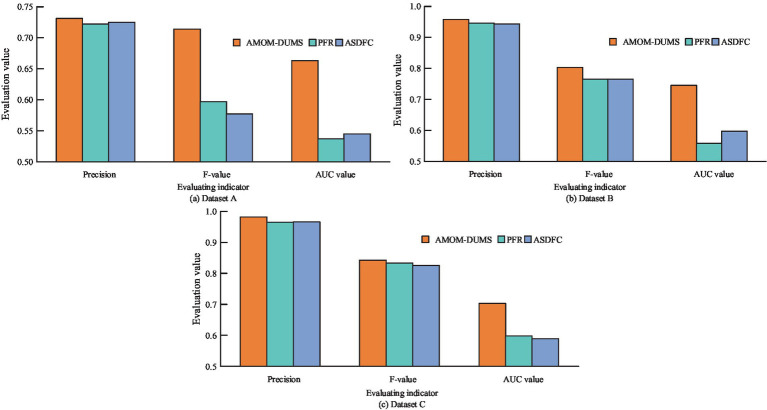
Performance results of different sampling algorithms on various datasets: **(a)** Dataset A; **(b)** Dataset B; **(c)** Dataset C.

In order to test the performance of AMOM-DUMS algorithm, several evaluation metrics like accuracy, precision, recall, *F*-score & AUC values are selected for evaluation. The study uses the current mainstream algorithms for comparative trials, i.e., particle filter resampling (PFR) algorithm and AC sampling during frequency changes (ACSDFC) in order to validate the performance of the intelligent sampling algorithm suggested by the study. The performance results of different sampling algorithms for each dataset is shown in Figure 7.

**Figure 8:**
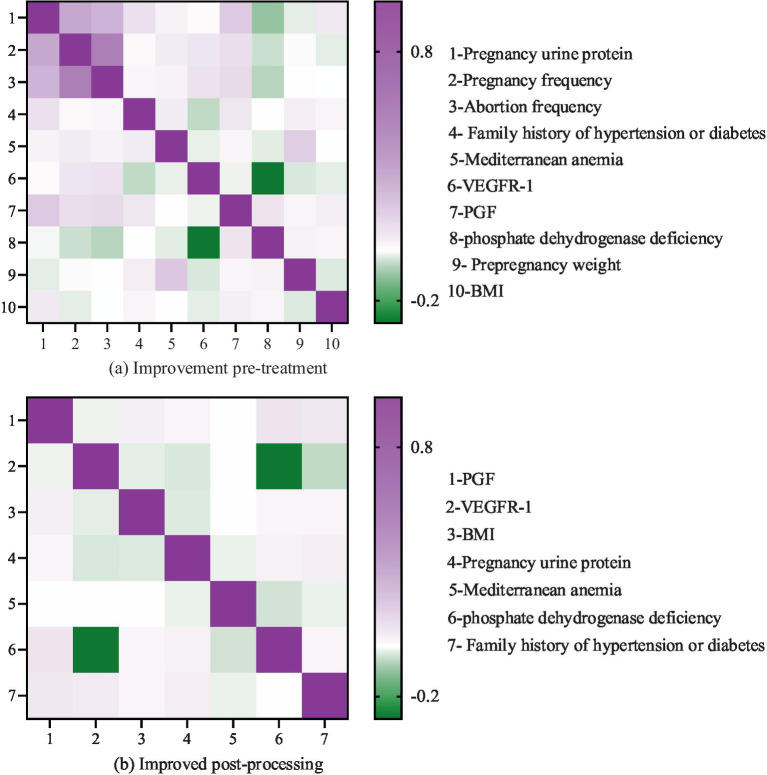
Comparison of feature correlation results before and after improving the intelligent data feature selection method optimized by *F*-Score: **(a)** Before treatment; **(b)** After treatment.

Figures 7a–c shows the performance of different sampling algorithms in dataset A, B and C, respectively. In Figure 7, in the three sub-datasets of the PIH dataset, the metrics of the proposed AMOM-DUMS algorithm are the best, and the accuracy, AUC value and *F*-score in dataset A are 0.731, 0.663, and 0.714, respectively. In dataset B, the metrics are 0.958, 0.745, and 0.803, respectively. The metrics in dataset C are 0.982, 0.703, and 0.842. The AUC values of the PFR algorithm in both datasets A and B are the lowest, 0.537 and 0.558, respectively, which indicates that the algorithm is less effective in dealing with the problem of data classification imbalance, while the AUC value of ACSDFC in dataset C is the smallest, 0.589. The aforementioned findings suggest that the study’s intelligent sampling method can greatly address the issue of healthcare data classification imbalance with a better classification effect. To more intuitively display the effect of the *F*-score optimization of intelligent data feature selection (IDFS) method after processing, the study uses heat map to process the correlation between variables, and the comparison of feature correlation results processed before and after the improvement of the *F*-score optimization of IDFS method can be obtained, as shown in Figure 8.

It is evident that prior to the enhancement (refer to Figure 8a), the heat map displayed on the left side of Figure 8 illustrates the feature correlation outcomes before the refinement of the IDFS method. In this heat map, the x-axis and y-axis denote the various features within the dataset, while the color of each cell signifies the correlation between the two features. The color bar located on the right side of the heat map represents the correlation coefficient, where darker shades indicate a stronger correlation, and lighter shades suggest a weaker correlation. The heat map reveals a significant number of features that exhibit high correlation with one another, suggesting the presence of redundancy within the dataset. For instance, the features “number of pregnancies,” “number of miscarriages,” “pre-pregnancy weight,” “number of births,” and “comorbid obesity” demonstrate a strong correlation among themselves.

The heat map displayed on the right side of Figure 8 illustrates the results of feature correlation after the enhancement of the IDFS method. This heat map reveals a significant reduction in the number of highly correlated features, suggesting that the IDFS method has effectively eliminated redundant features from the dataset. The remaining features, including “placental growth factor (PGF),” “vascular endothelial growth factor receptor (VEGFR-1),” “BMI,” “urinary protein in pregnancy,” “Mediterranean anemia,” “phosphate dehydrogenase deficiency,” and “family history of hypertension or diabetes,” exhibit low correlation with one another, signifying that they are more independent and pertinent to predicting PIH.

**Figure 9:**
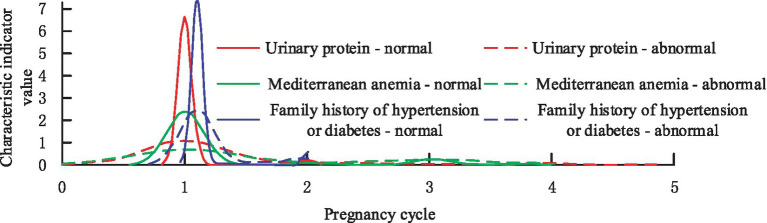
The distribution of optimal features in the PIH disease label section.

Three characteristics of Urinary protein in pregnancy, Mediterranean anemia, and family history of hypertension or diabetes are selected to demonstrate the distribution, as shown in Figure 9.

**Figure 10:**
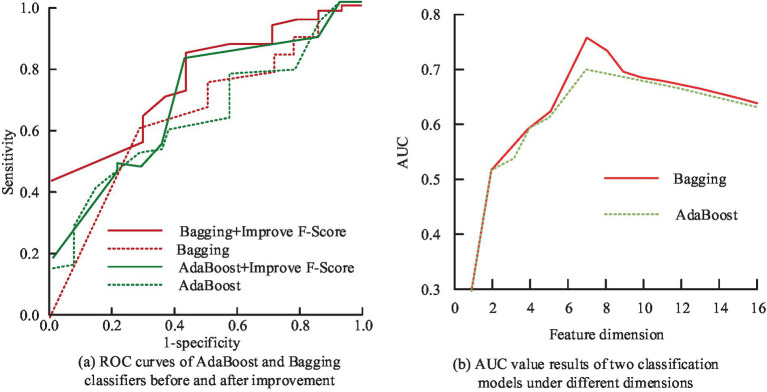
The AUC value results of AdaBoost and bagging classifiers before and after improving the intelligent data feature selection method optimized by *F*-Score: **(a)** ROC curves of AdaBoost and Bagging classifiers before and after improvement; **(b)** AUC value results of two classification models under different dimensions.

From Figure 9, it can be found that the features screened by the study before the mid-pregnancy are able to distinguish the diseased population well in terms of diagnosis, which is extremely important for the application of imbalance classification and prediction of medical data. Finally, the study explores the contribution that *F*-score optimized IDFS method to compare the performance of AdaBoost vs. bagging classifiers. The study evaluates the performance of the classifiers before and after the improvement using AUC values, and the results are shown in Figure 10.

Figure 10a shows the ROC curves of AdaBoost and bagging classifiers before and after the improvement that the *F*-score optimized IDFS method, and Figure 10b shows the results of the AUC values of the two classification models in different dimensions. In Figure 10, after removing redundant features using the *F*-score that optimized IDFS method, the AUC values of AdaBoost and Bagging increased from 0.651 to 0.787 and 0.645 to 0.714, respectively, in the pre-improvement treatment. At the same time, when the feature dimension is less, there will be a situation where important feature information is lost, so the AUC values of the two classification models are lower, both within 0.6. In addition, when the feature dimension is larger, the AUC values of the two classification models also appear to be reduced, because there are more irrelevant or redundant features. When the feature dimension is seven, the AUC value of AdaBoost and Bagging is the largest, which is consistent with the number of feature combinations processed by the IDFS method that was optimized by *F*-score. This suggests that the FS technique put out in the study may successfully choose the ideal feature combinations to guarantee the prediction model’s optimum performance.

### 4.2 Results based on IPIMHL-GA modeling

**Figure 11:**
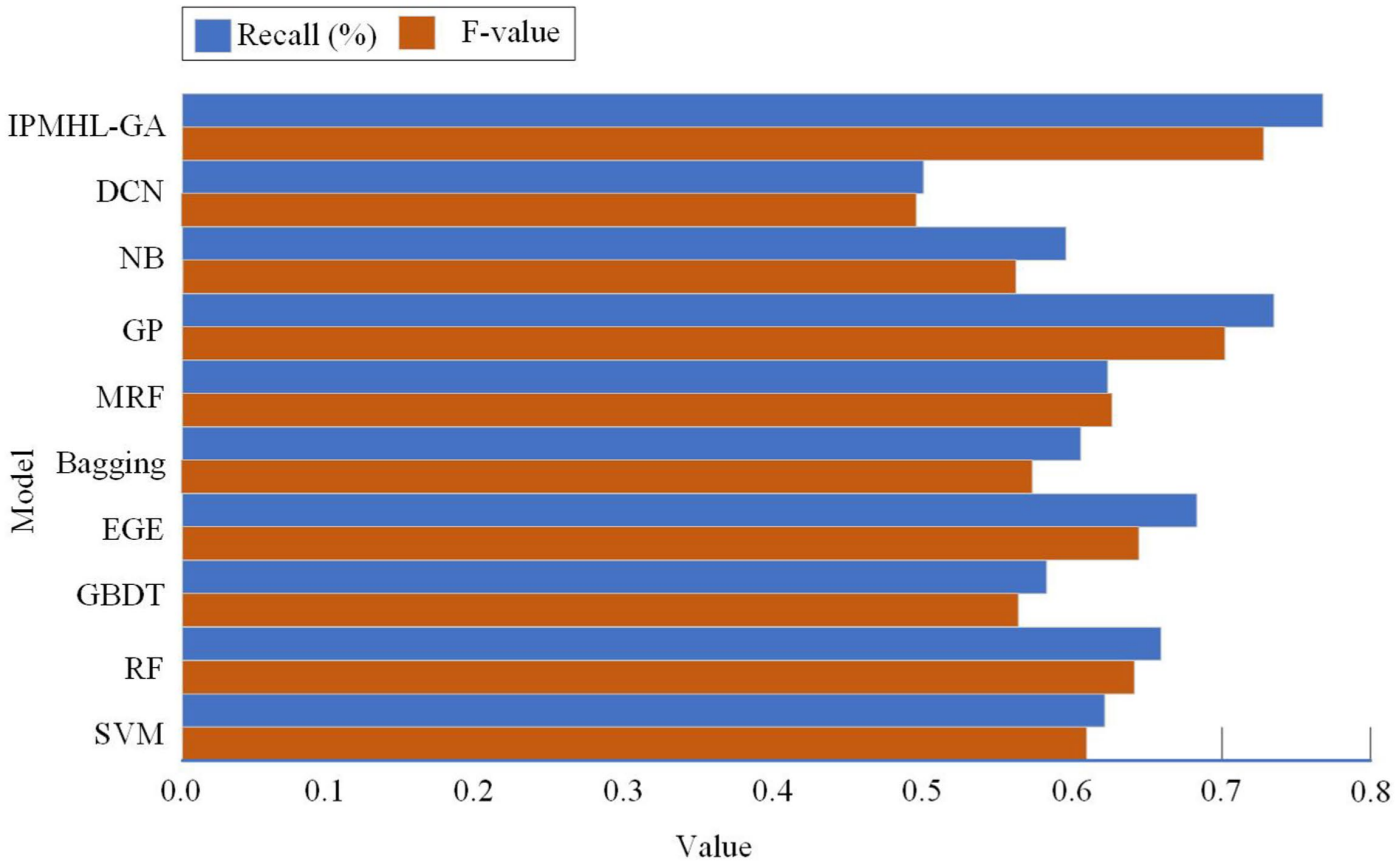
Prediction performance results of different classification models: **(a)** Recall; **(b)** *F*-value.

To validate the validity and feasibility of the IPIMHL-GA model proposed in the study, the study is evaluated using the recall rate, *F*-score, and AUC value, where the recall rate reflects the percentage of all true PIH-free patients that are correctly predicted and is used to categorize the model’s ability to detect positive cases. On the other hand, the *F*-score, which is a weighted and reconciled average of recall and accuracy, can assess how well a model performs in categories with limited sample sizes. To provide a more realistic validation of the models’ performance, the study compares the models with the most popular machine learning techniques currently in use, including SVM, bagging, RF, Markov random field (MRF), deep confidence network (DCN), gradient boosting decision tree (GBDT), NB, extreme gradient enhancement (EGE), and GP. The prediction performance results of different classification models are shown in Figure 11.

**Figure 12:**
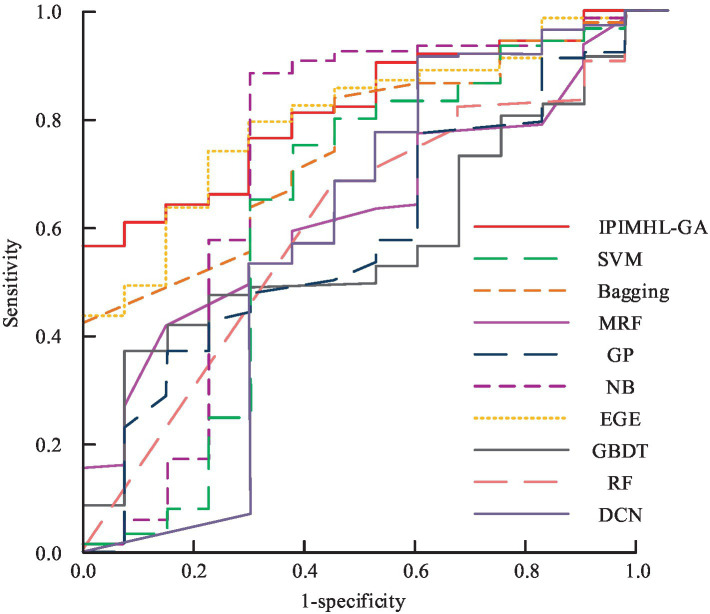
Prediction results of the area under the curve values for different classification models.

Figure 11 show the recall and *F*-score results of different classification models, respectively. In Figure 11, in the recall results, the IPIMHL-GA model is the best with the value of 0.768, while the GP model has the second-best performance with the value of 0.735, and the recall of the remaining classification models ranges from 0.499 to 0.683. In the results of *F*-score, the IPIMHL-GA model is still the best with the value of 0.728, while the GP model is still the second best with the value of 0.702, and the *F*-score of the remaining classification models ranges from 0.495 to 0.644. Figure 12 displays the anticipated AUC values for each of the categorization models.

In Figure 12, the AUC values of SVM model, bagging model, RF model, MRF model, DCN model, GBDT model, NB model, EGE model, GP model, and IPIMHL-GA model are 0.637, 0.755, 0.611, 0.635, 0.513, 0.594, 0.596, 0.725, 0.807, and 0.832, respectively. In conclusion, the intelligent PIH diagnosis and prediction method proposed in the study exhibits excellent performance and is suitable for application scenarios with high demand for real-time analysis. Furthermore, it effectively identifies and predicts PIH, provides reliable decision-making support for relevant personnel, and serves as a reference for the application of intelligent methods in OGT.

### 4.3 Clinical application of intelligent PIH diagnosis and prediction methods

**Figure 13:**
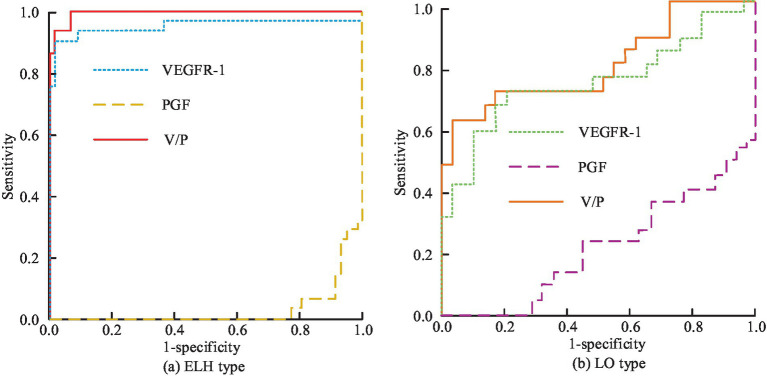
Comparison of ROC results in intelligent clinical diagnosis of various characteristic indicators under different types of disease in Group Z: **(a)** EH type; **(b)** LO type.

To further investigate the effectiveness of the research-designed intelligent PIH diagnosis and prediction method in clinical practice, 108 patients with preeclampsia at the onset of hospitalization are collected as Z group. In addition, the 34th gestational week is taken as the node, before that it was noted as early-onset (EO) and after that as late-onset (LO). In addition, the top two ranked PGF and VEGFR-1 after processing are analyzed using *F*-score that optimized IDFS method; in addition, the study introduced the ratio of VEGFR-1 to PGF, i.e., V/P for diagnosis and prediction. ROC comparison of intelligent clinical diagnosis results for each characterization index under Z-group for different pathogenesis types is shown in Figure 13.

**Figure 14:**
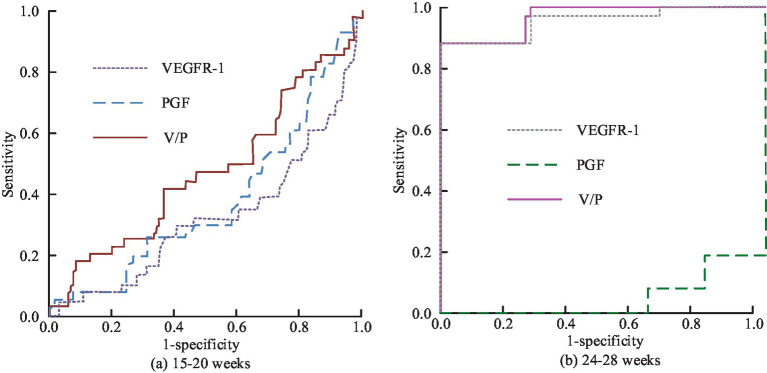
Prediction of ROC changes in preeclampsia by various characteristic indicators of Group Z at different gestational weeks: **(a)** Weeks 15-20; **(b)** Weeks 24-28.

Figures 13a,b show the intelligent clinical diagnosis of ROC results for each characterization index of EO type and LO type in group Z, respectively. In Figure 13, in the EO-Z group, the AUC value of single use of VEGFR-1 for diagnosis of EO type preeclampsia is 0.948, which corresponded to the highest diagnostic specificity and sensitivity of 91.1 and 93.5%, respectively, if the optimal cut-off value is 2,805 pg/mL. The diagnostic effect of using PGF alone is poor, with an AUC value of only 0.031. When the V/P values are used for diagnostic purposes, the AUC values are as high as 0.996, which corresponds to the highest diagnostic specificity and sensitivity when the optimal cutoff value is 27.25, which is 98.4 and 93.1%, respectively. In the LO-Z group, the AUC values of diagnosis using VEGFR-1 or PGF alone are 0.732 and 0.212, respectively, while the AUC value of V/P value is the highest at 0.792. According to the previous results, the diagnostic value of VEGFR-1, PGF, and V/P value characterization indexes is more obvious in weeks 15–20 versus weeks 24–28. Therefore, the study is predicted using each characterization index in weeks 15–20 and 24–28, and the predictive value results could be obtained, as shown in Figure 14.

Figures 14a,b correspond to the comparison of the results of the predictive value of each characteristic indicator at weeks 15–20 versus weeks 24–28. Figure 14 shows that by comparing the results of different indicators at weeks 15–20, the AUC values corresponding to VEGFR-1, PGF, and V/P values are 0.322, 0.375 and 0.465, respectively, indicating that the indicators are all poorly diagnosed. In the comparison of the results of the different indicators at 24–28 weeks, the AUC value for the prediction using only PGF alone is poor that has the value of 0.074. In contrast, the AUC value for the single application of VEGFR-1 is 0.948, which corresponded to an optimal cut-off value of 2745.40 pg/mL when the specificity and sensitivity of the prediction corresponded to 99.2 and 86.3%, respectively. The AUC of V/P value is 0.953, while the specificity and sensitivity of prediction corresponded to 99.2 and 86.3% when the best cut value is at 11.37 pg/mL. In conclusion, the intelligent PIH diagnosis and prediction method proposed in the study has good results in clinical practice and can be used for the promotion of OGT.

The results indicated that IPIMHL-GA surpassed all these methods in terms of recall, *F*-score, and AUC, achieving a recall of 0.768, an *F*-score of 0.728, and an AUC of 0.832. In comparison, Li’s et al. method recorded values of 0.723, 0.693, and 0.789; Wang’s et al. method yielded the values of 0.736, 0.706, and 0.794; Kumar’s et al. method produced the values of 0.729, 0.694, and 0.785; and Chen’s et al. method resulted in the values of 0.741, 0.705, and 0.796. These results imply that IPIMHL-GA is a superior approach for predicting PIH, likely attributable to its effective integration of feature selection and classification algorithms, which enhances the identification of pertinent features and strengthens the classification model. Furthermore, the results underscored the significance of employing a combination of feature selection and classification algorithms in predicting PIH, as this strategy aided in pinpointing the most relevant features and enhanced the prediction model’s accuracy.

The investigation into generative and deep learning models for predicting PIH uncovered numerous opportunities to enhance the precision and efficacy of these models. Generative models, including generative adversarial networks (GANs) and variational autoencoders (VAEs), are capable of producing new data samples that closely resemble the training data, thereby facilitating the prediction of PIH by generating samples that are likely to exhibit this condition. Deep learning architectures, such as convolutional neural networks (CNNs), recurrent neural networks (RNNs), and long short-term memory (LSTM) networks, possess the ability to identify intricate patterns within data that signify PIH, resulting in heightened accuracy. Furthermore, hybrid models, like GAN-VAE and CNN-LSTM, can merge the advantages of various models to enhance overall performance.

The benefits of employing generative and deep learning models for PIH prediction encompass increased accuracy, the capability to manage high-dimensional data, and the potential to learn from limited datasets. Nonetheless, challenges such as interpretability, overfitting, and data quality must be tackled. Prospective avenues for advancing the use of generative and deep learning models in PIH prediction include the creation of more interpretable models, enhancement of data quality, and the application of transfer learning. By capitalizing on these models and addressing their associated challenges, researchers and healthcare professionals can significantly improve the accuracy and effectiveness of PIH predictions, ultimately contributing to better patient outcomes.

## 5 Conclusion

Aiming at the imbalance problem of medical data classification, the study took three perspectives of sampling, including FS and predictive model as the entry point, proposed AMOM-DUMS sampling algorithm and intelligent medical data FS method based on *F*-score optimization, and designed IPIMHL-GA model. The experimental results revealed that in the recall results, the IPIMHL-GA model was the best with the value of 0.768, while the GP model had the second-best performance with the value of 0.735, and the recall of the remaining classification models was in the range of 0.499 and 0.683. Concerning the results of *F*-score, the IPIMHL-GA model was still the best with the value of 0.728, while the GP model still had the second-best performance with the value of 0.702, and the *F*-score of the remaining classification models ranged from 0.495 to 0.644. The AUC values of the SVM model, bagging model, RF model, MRF model, DCN model, GBDT model, NB model, EGE model, GP model, and the IPIMHL-GA model were, in turn, 0.637, 0.755, 0.611, 0.635, 0.513, 0.594, 0.596, 0.725, 0.807, and 0.832. Finally, in the results of clinical trials, the AUC value for diagnosis of EO-type preeclampsia using VEGFR-1 alone in the EO-Z group was 0.948. Diagnosis using PGF alone was poorer, with an AUC value of 0.031. In contrast, the AUC value for diagnosis by the V/P value was as high as 0.996. In the LO-Z group, the AUC values for diagnosis by using VEGFR-1 or PGF alone were 0.732 versus 0.212, respectively, while the AUC value for V/P values was as high as 0.792. In summary, the method proposed in the study could solve the noisy data during medical imbalance data sampling, improve the classification ability of the model, realize real-time accurate ID and prediction, and provide a convenient ID and prediction method for OGT. However, the study still had deficiencies, and more detailed values of the feature variables could be provided for reference in future studies. Furthermore, the system could be configured to issue a risk warning prompt when a diagnostic feature reached a threshold value. It enabled physicians to make diagnoses more efficiently and accurately, while also facilitating the development of diagnostic protocols for patients. Additionally, educators could utilize OBGYN intelligent methods in the classroom with greater ease.

## Data Availability

The original contributions presented in the study are included in the article/supplementary material, further inquiries can be directed to the corresponding author.
